# Trends in Use of Prescription Nonsteroidal Anti-inflammatory Medications Before vs After Implementation of a Florida Law Restricting Opioid Prescribing for Acute Pain

**DOI:** 10.1001/jamanetworkopen.2021.13383

**Published:** 2021-06-11

**Authors:** Shailina Keshwani, Ivanna Grande, Michael Maguire, Amie Goodin, Scott M. Vouri, Juan M. Hincapie-Castillo

**Affiliations:** 1 Department of Pharmaceutical Outcomes & Policy, University of Florida, Gainesville; 2 Center for Drug Evaluation and Safety, University of Florida, Gainesville; 3 UF Health Physicians, Gainesville, Florida; 4 Pain Research and Intervention Center of Excellence, University of Florida, Gainesville

## Abstract

**Importance:**

Previous research has shown an immediate reduction in new opioid users and use after implementation of the opioid supply restriction laws. Assessment of the association between opioid restrictions and alternative treatment options, such as nonsteroidal anti-inflammatory drugs (NSAIDs), is needed to evaluate potential unintended consequences for patients requiring analgesia.

**Objective:**

To evaluate the association between an opioid restriction law in Florida and use of prescription NSAIDs.

**Design, Setting, and Participants:**

This quality improvement study used interrupted time series analyses accounting for autocorrelation to estimate immediate and trend changes in the prescribing and use of prescription NSAIDs in Florida before and after implementation of a state law limiting opioid prescriptions to a 3-day supply. Participants were enrollees in a single private health plan of a large university and health system employer in Florida from January 2015 to June 2019.

**Exposures:**

Prescriptions for NSAIDs, ascertained from pharmacy claims data.

**Main Outcomes and Measures:**

The following outcomes were calculated monthly per 1000 plan enrollees: (1) number of NSAID users; (2) mean days’ supply of NSAIDs per prescription; and (3) mean number of NSAID prescriptions. Individuals were classified as NSAID users if they had at least 1 NSAID prescription in a given month. Analysis was stratified by route of NSAID administration (oral or nonoral).

**Results:**

Among 46 783 NSAID users with 79 089 NSAID prescriptions during the study period, the median age was 47 years (interquartile range, 35-57 years). After implementation of the opioid restriction law, the number of NSAID users immediately increased, but the difference was not significant (change, 0.82 per 1000 patients; 95% CI, −0.67 to 2.30 per 1000 patients). No significant change in the days’ supply of oral NSAID users occurred (change, 0.21 days per prescription; 95% CI, −1.66 to 2.08 days per prescription). Before implementation of the law, there was a nonsignificant decreasing trend in NSAID prescriptions (rate of change, −0.03 per month per 1000 enrollees; 95% CI, −0.13 to 0.07 per month per 1000 enrollees; after implementation, there was a nonsignificant increase in the number of oral and nonoral NSAID prescriptions (change, 1.49 per 1000 enrollees; 95% CI, −3.38 to 6.37 per 1000 enrollees).

**Conclusions and Relevance:**

In this quality improvement study, prescribing and use of prescription NSAIDs did not increase after implementation of a law restricting opioid analgesic prescriptions in Florida. These findings suggest possible greater use of over-the-counter NSAIDs after implementation of the law, but further research is needed to evaluate changes in the use of nonopioid analgesics and alternative pain therapies.

## Key Points

**Question:**

Was implementation of a Florida law restricting the prescription of opioids associated with changes in the prescribing and use of prescription nonsteroidal anti-inflammatory drugs (NSAIDs)?

**Findings:**

In this quality improvement study of 79 089 prescription claims from a private health plan, implementation of an opioid restriction law in Florida was not associated with a significant increase in the number of prescription NSAID users, the number of NSAID prescriptions, or the number of days’ supply of oral NSAIDs per prescription dispensed.

**Meaning:**

In this study, after the implementation of a law restricting opioid prescriptions in Florida, there was not a corresponding increase in prescribing or use of nonopioid NSAID analgesic medications.

## Teaser

This quality improvement study examines changes in the prescribing and use of prescription nonsteroidal anti-inflammatory drugs (NSAIDs) after implementation of a law restricting the prescription of opioids in Florida.

## Introduction

In light of continued increases in opioid-related overdose deaths in the US, many state governments have enacted legislation intended to reduce the opioid prescription supply. Recent data^1^ show that most opioid-related deaths are associated with use of nonprescription opioids (ie, illicit sources such as fentanyl and heroin products); however, more than half of all states have enacted legislation to restrict opioid prescribing or dispensing for acute pain indications.^2^ Many states have adopted legislation that restricts first-time opioid prescriptions to a certain number of days’ supply or the total morphine milligram equivalent of the prescription. The laws vary from state to state, and Tennessee, Kentucky, and Florida currently have the strictest policies.^3^

On July 1, 2018, Florida implemented House Bill 21 (HB21), which limited the prescription of a Schedule II controlled substance to a 3-day supply or, in certain cases, a 7-day supply.^4^ Previous research^5,6,7^ has shown that the number of days’ supply per prescription and first-time opioid use decreased after implementation of the law, leading to a lower supply of opioids in the state, as intended by the bill. A retrospective study^8^ evaluating prescription patterns for acute pain after surgery found no increase in the percentage of patients visiting the emergency department postoperatively for pain control immediately after the law was implemented, but limited evidence is available to examine pain management for other conditions after opioid restrictions. Therefore, it is still unclear whether prescription opioid restriction legislation has unintended consequences for patients requiring analgesics.

Given the increase in policy efforts to decrease opioid prescribing, assessment of the associations opioid restriction laws could have with the use of nonopioid analgesics such as nonsteroidal anti-inflammatory drugs (NSAIDs) is needed. NSAIDs are common first-line agents used for mild to moderate acute pain, and although they are generally considered to be safe and effective over-the-counter (OTC) medications, their use is associated with complications affecting the gastrointestinal tract, cardiovascular system, and kidneys.^9,10^ A previous study^11^ conducted using the Veterans Health Administration database showed no change in NSAID use from 2011 to 2015 before vs after the implementation of the Opioid Safety Initiative. However, to our knowledge, there are no data to date showing unintended changes in trends of NSAID use associated with state implementation of opioid prescribing limits or supply-restriction limits. To fully assess the intended and unintended consequences for patients with pain, research is needed to address medication use not limited to opioid prescriptions. The objective of this study was to assess the association between the implementation of Florida HB21 with trends in NSAID prescribing and use among a group of privately insured patients.

## Methods

### Study Design and Sample

This quality improvement study used a quasi-experimental design with interrupted time series models to evaluate trend and level changes associated with policy implementation. We analyzed pharmacy prescription claims for NSAIDs from a single health plan that serves more than 45 000 employees of a large university and health system employer in Florida. We analyzed data from January 1, 2015, through June 30, 2019. Because HB21 was implemented on July 1, 2018, the preimplementation period ranged from January 1, 2015, to June 30, 2018, and the postimplementation period ranged from July 1, 2018, to June 30, 2019. There were no exclusions based on age. Patients were required to have at least 1 NSAID prescription to be considered an NSAID user in a given month. This study was approved by the University of Florida institutional review board and was exempted from informed consent requirements because data were deidentified. The study followed the Standards for Quality Improvement Reporting Excellence (SQUIRE) reporting guideline.

### Medication Measures

We identified NSAID prescriptions using generic ingredient drug names (eg, ibuprofen, indomethacin) and included single and combination products of NSAIDs. We excluded records for ophthalmic formulations (eg, ketorolac ophthalmic solution, flurbiprofen eye drops) and combination products containing NSAIDs with opioids (eg, hydrocodone with ibuprofen). Pharmacy records for returned NSAID prescriptions were excluded from the analysis when calculating the prevalence of NSAID use among plan enrollees and the mean number of days’ supply of NSAID prescriptions dispensed. However, these records were retained for the analysis when evaluating the mean number of NSAID prescriptions per month. Our analysis on number of prescriptions and number of users was stratified by the route of administration of the NSAID product.

### Outcomes

We calculated (1) the mean number of NSAID prescriptions per 1000 plan enrollees by month (total number of NSAID prescriptions each month/total number of patients enrolled in that month), (2) the prevalence of NSAID users per 1000 plan enrollees by month (total distinct patients prescribed at least 1 NSAID each month/total number of patients enrolled in that month), and (3) the mean number of days’ supply of oral NSAIDs per month. For the outcome of days’ supply, we analyzed only oral NSAIDs owing to the unreliable data on days’ supply for nonoral NSAID formulations (eg, diclofenac gel).^12^

### Statistical Analysis

Descriptive statistics were used to report demographic data. Our 3 outcomes of interest were analyzed using interrupted time series models, which accounted for autocorrelation of error terms. Interrupted time series is the preferred quasi-experimental study design for policy analyses because it allows for an assessment of preexisting trends in the data before policy implementation (ie, time effect) and both immediate changes in the outcome (ie, level effect) and changes in the outcome trend (ie, trend effect) after implementation.^13^ We used R statistical software, version 4.0.3 (R Project for Statistical Computing), to fit a generalized least-squares linear model with an autoregressive moving average correlation structure of order (*p*, *q*). The autocorrelation function and partial autocorrelation function were analyzed to obtain the *p* and *q* parameters. The model coefficients for time effect, level, and trend change owing to policy intervention were evaluated. A 2-sided *P* < .05 was considered statistically significant. We conducted a sensitivity analysis introducing a 2-month phase-in period after implementation of HB21 to assess the robustness of our estimates. Data analysis was conducted using R, version 4.0.3, and SAS, version 9.4 (SAS Institute Inc).

## Results

A total of 79 089 NSAID prescription claims were recorded during the study period. Of these, 15 646 prescription records were returned (ie, the prescription was filled by the pharmacy but was not ultimately dispensed to the patient); therefore, 62 125 prescriptions were analyzed for the outcome of NSAID users and mean days’ supply after removing 1318 ophthalmic formulations and combination products containing NSAIDs and opioids. Of the 46 783 unique or individual NSAID users throughout the study period, 42 624 were unique or individual users of oral NSAIDs and 5138 were unique or individual users of nonoral NSAIDs.

The median age of all NSAID users was 47 years (interquartile range, 35-57 years). After applying the exclusion criteria for 1318 ophthalmic formulations and combination products containing NSAIDs and opioids (2% of the total prescriptions), there were a total of 77 771 NSAID prescription records. Of these, 68 117 (88%) were prescriptions for oral NSAIDs and 9654 (12%) were prescriptions for nonoral NSAIDs. The most commonly prescribed oral NSAIDs were ibuprofen, which accounted for 22 479 oral prescriptions (33%), followed by meloxicam (17 710 [26%]), naproxen (10 218 [15%]), and others (17 710 [26%]). The most prescribed nonoral NSAIDs were diclofenac sodium, which accounted for 8978 nonoral prescriptions (93%), followed by diclofenac epolamine (290 [3%]) and others (386 [4%]).

### Number of NSAID Prescriptions

**Figure 1.**
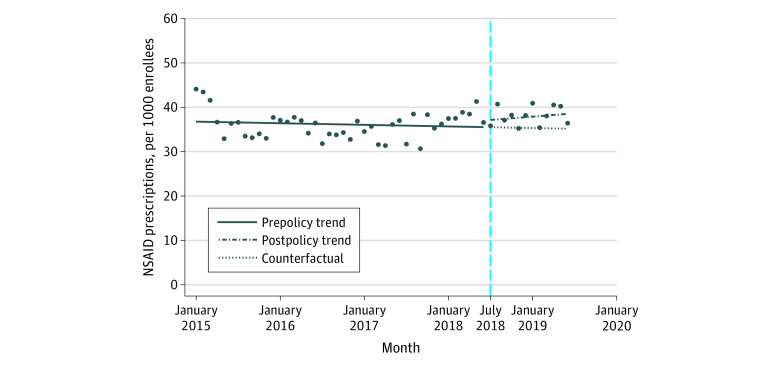
Trends in the Number of All Nonsteroidal Anti-inflammatory Drug (NSAID) Prescriptions per Month, January 2015 to June 2019 Markers indicate mean values. The vertical dashed line indicates implementation of Florida House Bill 21.

**Figure 2.**
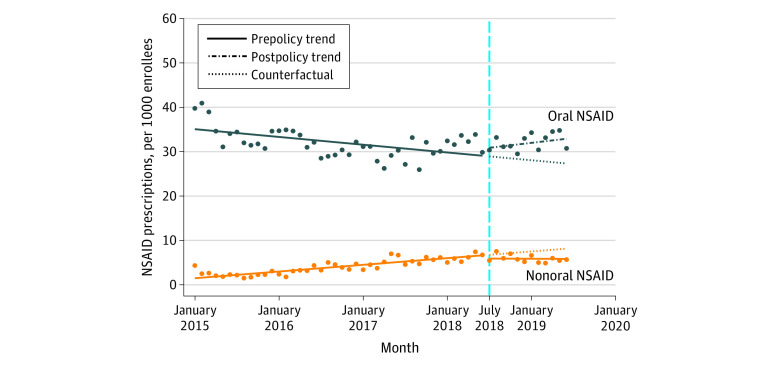
Trends in the Number of Nonsteroidal Anti-inflammatory Drug (NSAID) Prescriptions by Route of Administration, January 2015 to June 2019 Markers indicate mean values. The vertical dashed line indicates implementation of Florida House Bill 21.

After the implementation of HB21, the number of all NSAID prescriptions dispensed immediately increased, but the difference was not significant (change, 1.49 per 1000 enrollees; 95% CI, −3.38 to 6.37 per 1000 enrollees) (Table). Before implementation of HB21, there were nonsignificant decreasing trends in all NSAID prescriptions (rate of change, −0.03 per month per 1000 enrollees; 95% CI, −0.13 to 0.07 per month per 1000 enrollees). After implementation of the policy, there were nonsignificant increasing trends in all NSAID prescriptions (rate of change, 0.15 per month per 1000 enrollees; 95% CI, −0.47 to 0.77 per month per 1000 enrollees) (Figure 1). In the analysis stratified by route of administration, implementation of HB21 was associated with an immediate but nonsignificant increase in the number of prescriptions of oral NSAIDs (change, 1.61 per 1000 enrollees; 95% CI, −2.80 to 6.02 per 1000 enrollees) and a nonsignificant increasing trend in prescriptions of oral NSAIDs (rate of change, 0.33 per month per 1000 enrollees; 95% CI, −0.25 to 0.91 per month per 1000 enrollees) (Figure 2). After the policy implementation, there was a nonsignificant decrease in the number of prescriptions of nonoral NSAIDs (change, −0.72 per 1000 enrollees; 95% CI, −1.88 to 0.44 per 1000 enrollees) and a nonsignificant decreasing trend in prescriptions of nonoral NSAIDs (rate of change, −0.13 per month per 1000 enrollees; 95% CI, −0.29 to 0.02 per month per 1000 enrollees) (Figure 2).

**Table.** Coefficients From Interrupted Time Series Models Using Monthly Autoregressive Moving Averages

Analysis	Intercept (95% CI)	Time (95% CI)	Level (95% CI)	Trend (95% CI)
NSAID prescriptions per 1000 enrollees				
All	36.80 (34.28 to 39.32)	−0.03 (−0.13 to 0.07)	1.49 (−3.38 to 6.37)	0.15 (−0.47 to 0.77)
Oral	35.22 (32.74 to 37.69)	−0.15 (−0.25 to −0.06)	1.61 (−2.80 to 6.01)	0.33 (−0.25 to 0.91)
Nonoral	1.36 (0.99 to 1.72)	0.13 (0.11 to 0.14)	−0.72 (−1.88 to 0.44)	−0.13 (−0.29 to 0.02)
NSAID users per 1000 enrollees				
All	22.62 (21.94 to 23.30)	−0.03 (−0.06 to −0.01)	0.82 (−0.67 to 2.30)	0.06 (−0.13 to 0.24)
Oral	21.96 (21.30 to 22.61)	−0.08 (−0.11 to −0.06)	1.12 (−0.32 to 2.56)	0.10 (−0.08 to 0.28)
Nonoral	0.80 (0.66 to 0.95)	0.06 (0.06 to 0.07)	−0.26 (−0.67 to 0.16)	−0.06 (−0.11 to 0.00)
Oral NSAID supply	25.90 (23.64 to 28.16)	0.10 (0.02 to 0.19)	0.21 (−1.66 to 2.08)	−0.09 (−0.40 to 0.22)

Abbreviation: NSAID, nonsteroidal anti-inflammatory drug.

### Number of NSAID Users

**Figure 3.**
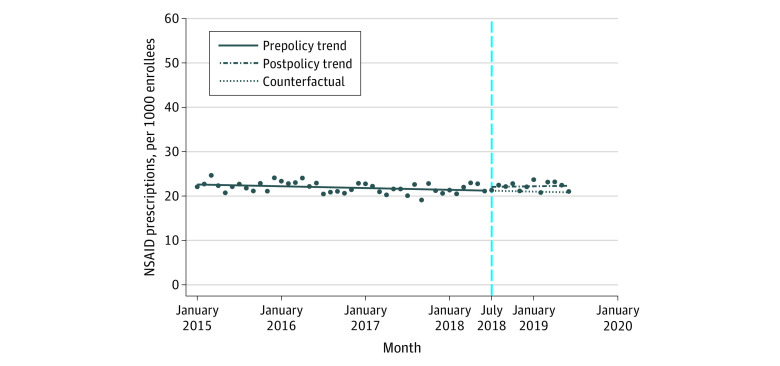
Trends in the Prevalence of Nonsteroidal Anti-inflammatory Drug (NSAID) Use per Month Among Plan Enrollees, January 2015 to June 2019 Markers indicate mean values. The vertical dashed line indicates implementation of Florida House Bill 21.

**Figure 4.**
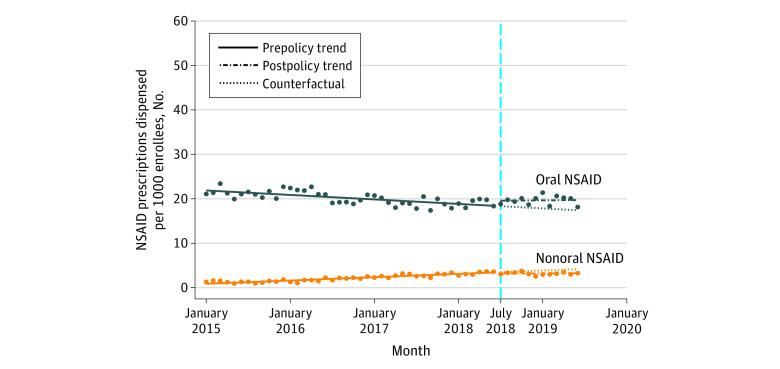
Trends in Nonsteroidal Anti-inflammatory Drug (NSAID) Use per Month Among Plan Enrollees by Route of Administration, January 2015 to June 2019 Markers indicate mean values. The vertical dashed line indicates implementation of Florida House Bill 21.

After the implementation of HB21, the number of users of all NSAIDs immediately increased, but the difference was not statistically significant (change, 0.82 per 1000 patients; 95% CI, −0.67 to 2.30 per 1000 patients) (Table). Before implementation of HB21, there were significant decreasing trends in the number of users for all NSAIDs (rate of change, −0.03 per month per 1000 enrollees; 95% CI, −0.06 to −0.01 per month per 1000 enrollees) (Figure 3). After implementation of HB21, there was an immediate but nonsignificant increase in the number of users of oral NSAIDs (change, 1.12 per 1000 enrollees; 95% CI, −0.32 to 2.56 per 1000 enrollees) and a decrease in the number of users of nonoral NSAIDs (change, −0.26 per 1000 enrollees; 95% CI, −0.67 of 0.16 per 1000 enrollees) (Figure 4). Before implementation of HB21, there was a significant decreasing trend in the number of users of oral NSAIDs (rate of change, −0.08 per month per 1000 enrollees; 95% CI, −0.11 to −0.06 per month per 1000 enrollees) (Figure 4), which did not change significantly in the period after the policy implementation. Before implementation of HB21, there was a significant increasing trend in the number of users of nonoral NSAIDs (rate of change, 0.06 per month per 1000 enrollees; 95% CI, 0.06-0.07 per month per 1000 enrollees) (Figure 4), which, similar to the trend for oral NSAIDs, did not change significantly in the postimplementation period.

### Number of Days’ Supply

After policy implementation, there was not an immediate significant change in the days’ supply for prescriptions of oral NSAIDs (change, 0.21 days per prescription; 95% CI, −1.66 to 2.08 days per prescription) (Table). Before implementation of HB21, there were significant increasing trends in days’ supply for prescriptions of oral NSAIDs (change, 0.10 days per prescription; 95% CI, 0.02-0.19 days per prescription) (eFigure in the Supplement). After policy implementation, there were nonsignificant decreasing trends in days’ supply for prescriptions of oral NSAIDs (change, −0.09 days per prescription; 95% CI, −0.40 to 0.22 days per prescription) (eFigure in the Supplement). The findings of the sensitivity analysis assessing the robustness of the results by including a 2-month phase-in period after the introduction of the law revealed effect estimates similar to those found in the primary analyses (eTable in the Supplement).

## Discussion

This study revealed no significant increases in the number of NSAID users, days’ supply per NSAID prescription, and mean number of NSAID prescriptions dispensed after the implementation of a Florida law limiting the prescription of opioids to a 3-day supply. Similar changes were observed for oral and nonoral NSAID formulations. To our knowledge, this is one of the first studies to investigate the association between opioid restriction legislation and use of prescription NSAIDs.

The immediate increases observed in NSAID use after implementation of HB21 may suggest that NSAIDs are a main alternative to opioid analgesic prescribing options, but the increases were not significant compared with the preexisting trends. Several possible explanations may account for the decreasing trend in the prescribing and use of nonoral NSAIDs found in this study, including insufficient sample size. Of the total number of NSAID prescription claims observed, prescriptions for oral NSAIDs accounted for 88%, whereas prescriptions for nonoral NSAIDs accounted for 12%. This finding suggests that the sample size was not large enough to accurately examine the consequences of the policy change for nonoral route of administration. In addition, we speculated that prescriptions of nonoral NSAIDs, such as topical formulations that are nonformulary medications, may have limited the scope of our analysis of nonoral NSAID use.

As part of a continued effort to address the opioid crisis, the Florida legislature implemented HB451 on July 1, 2019, which requires prescribers to inform their patients of available nonopioid alternatives for the treatment of pain before initiating opioid therapy.^4^ In addition, health care professionals in Florida are required to discuss the advantages and disadvantages of nonopioid alternatives and provide a printed educational pamphlet created by the Florida Department of Health discussing nonopioid medications and therapies to reduce exposure to opioids and dependency.^4^ Because this law was implemented after our study period, we were unable to evaluate its association with NSAID use. Although the current study showed a nonsignificant change in use of prescription NSAIDs after implementation of HB21, it is possible that HB451 will be associated with an increase in the prevalence of NSAID use and other nonopioid alternatives by empowering patients to make informed decisions about a full range of therapies to manage their pain.

Despite our findings of nonsignificant increases in NSAID use, according to the US Centers for Disease Control and Prevention, the use of nonopioid prescription pain medications in the US increased from 2009 to 2018.^14^ Health care professionals may consider all available alternatives for pain treatment strategies. NSAIDs are considered an effective first-line therapy alternative to opioid medications for mild to moderate acute and chronic pain.^15^ Although NSAIDs are generally considered safe and are readily accessible over the counter to the public, there has been extensive reporting on the risks and safety issues associated with oral NSAIDs. Adverse effects of regular NSAID use include several different types of kidney injury and gastrointestinal bleeding.^16,17^ A previous study^18^ reported increased use of prescription analgesics including NSAIDs from 2006 to 2015 among Medicare patients with chronic kidney disease.^18^ Another study^19^ showed that adverse cardiovascular events were associated with selective cyclooxygenase 2 inhibitors and nonselective NSAIDs such as diclofenac. Thus, clinicians’ decisions to prescribe NSAIDs for acute and chronic conditions require careful consideration and patient-specific approaches to deliver safe and effective care. Although shifting from opioid analgesics to NSAIDs could be clinically warranted at times, this practice might lead to patient harm in the form of (1) greater toxic effects and adverse events owing to the requirement of high doses of NSAIDs to achieve desired analgesia or (2) uncontrolled pain owing to a lack of analgesia altogether with the choice of NSAID products.

As many states increase their focus on safer dose recommendations and prescribing restrictions to reduce the potential for opioid misuse and diversion, early evaluations of these and other related opioid restriction policies have not shown a substantial decrease in opioid prescribing and dose recommendation.^20^ A recent study by Chua and colleagues^20^ that analyzed early evidence of opioid prescribing limits showed modest reductions in opioid prescribing associated with opioid prescribing limits that were already below the level allowed, no required daily dose restrictions, and clinicians not complying with prescribing limits. Additional clinical perspectives should be considered when designing legislation for safe opioid prescribing to address best practices for general pain care. Further insight into changes in use of treatment alternatives such as gabapentinoids, muscle relaxants, and benzodiazepines as well as changes in illicit drug use warrants exploration in future studies.

### Limitations

This study has limitations. First, we analyzed only pharmacy prescription claims and did not capture use of OTC NSAIDs. Claims data can capture only those claims that are paid through insurance, and OTC NSAIDs are out-of-pocket costs; therefore, we were unable to assess trends in use of OTC NSAIDs. Based on the number of returned NSAID prescriptions, patients might have purchased NSAID medications as OTC products. Further research is necessary to understand the association of opioid-related legislation with trends and shifts in use of OTC NSAIDs. To capture the number of dispensed NSAID prescriptions, we excluded the returned NSAID prescriptions from the data analysis. Thus, the number of NSAID users may be underrepresented. In addition, the health plan database used for this study lacks information about indications for the corresponding prescriptions claims; therefore, we could not assess whether each NSAID prescription was an appropriate therapeutic option. Furthermore, we evaluated the mean days’ supply only for prescriptions of oral NSAIDs because information about days’ supply for nonoral formulations is usually overestimated from the actual days’ supply for the purpose of claims processing.^12^ It is also likely that there are other factors associated with NSAID prescribing in addition to prescription opioid policy changes. Our analysis was limited to a patient population that may be younger and healthier than the general population given that the patients were recipients of employer-sponsored insurance coverage at the time of the study; therefore, it is unclear whether the observed changes would be consistent in other populations. Nevertheless, the methods used in this study can be applied to evaluate the association between opioid restriction policies and NSAID use among publicly insured patients such as Medicare and Medicaid beneficiaries. Furthermore, this study could not assess long-term trend changes associated with policy implementation beyond the study period.

## Conclusions

In this quality improvement study, prescribing and use of prescription NSAIDs did not increase after implementation of Florida HB21, which restricted opioid analgesic prescriptions in the state. These findings suggest greater use of OTC NSAIDs after implementation of the law; thus, evaluation of OTC NSAID use and use of other nonopioid analgesics is warranted. Further research is also needed to evaluate changes in nonpharmacotherapies for pain management and their association with health outcomes in the wake of reduced opioid supply.

## Notes

Supplement. **eTable.** Sensitivity Analysis—Coefficients From Interrupted Time Series Models Using Autoregressive Moving Averages (Monthly) With Two-Month Phase-in Period **eFigure.** Number of Days’ Supply for Oral NSAID Users per Month
