# Supplementary material for: Trends in Use of Prescription Nonsteroidal Anti-inflammatory Medications Before vs After Implementation of a Florida Law Restricting Opioid Prescribing for Acute Pain
Source: JAMA Netw Open. 2021 Jun 11;4(6):e2113383. doi: 10.1001/jamanetworkopen.2021.13383 (PMC8196340; doi:10.1001/jamanetworkopen.2021.13383)
Supplement: Supplement. — eTable. Sensitivity Analysis—Coefficients From Interrupted Time Series Models Using Autoregressive Moving Averages (Monthly) With Two-Month Phase-in Period eFigure. Number of Days’ Supply for Oral NSAID Users per Month [file jamanetwopen-e2113383-s001.pdf]

Supplementary Online Content

Keshwani S, Grande I, Maguire M, Goodin A, Vouri SM, Hincapie-Castillo JM. Trends in use of prescription nonsteroidal anti-inflammatory medications before vs after implementation of a Florida law restricting opioid prescribing for acute pain. *JAMA Netw Open*. 2021;4(6):e2113383. doi:10.1001/ jamanetworkopen.2021.13383

eTable. Sensitivity Analysis—Coefficients From Interrupted Time Series Models Using Autoregressive Moving Averages (Monthly) With Two-Month Phase-in Period

eFigure. Number of Days' Supply for Oral NSAID Users per Month

This supplementary material has been provided by the authors to give readers additional information about their work.

© 2021 Keshwani S et al. *JAMA Network Open*.

eTable. Sensitivity Analysis—Coefficients From Interrupted Time Series Models Using Autoregressive Moving Averages (Monthly) With Two-Month Phase-in Period

**Analysis**	**NSAID Type**	**Intercept (95% CI)**	**Time (95% CI)**	**Level (95% CI)**	**Trend (95% CI)**
**Avg no. of NSAID prescription per 1,000 enrollees**	All	36.86 (34.27, 39.45)	-0.03 (-0.14, 0.07)	0.57 (-4.70, 5.84)	0.26 (-0.41, 0.92)
	Oral	35.25 (32.74, 37.76)	-0.15 (-0.25, -0.05)	1.13 (-3.50, 5.76)	0.39 (-0.22, 0.99)
	Non oral	1.38 (0.99, 1.77)	0.12 (0.12, 0.14)	-1.25 (-2.51, 0.01)	-0.08 (-0.24, 0.09)
**NSAID users per 1,000 enrollees**	All	22.62 (21.93, 23.31)	-0.03 (-0.06, -0.01)	0.63 (-1.22, 2.48)	0.08 (-0.14, 0.29)
	Oral	21.96 (21.28, 22.63)	-0.08 (-0.11, -0.06)	1.11 (-0.68, 2.90)	0.10 (-0.11, 0.31)
	Non oral	0.81 (0.66, 0.96)	0.06 (0.06, 0.07)	-0.27 (-0.70, 0.17)	-0.05 (-0.11, 0.00)
**Mean no. of Days'supply**	Oral	25.90 (23.64, 28.16)	0.10 (0.02, 0.19)	0.21 (-1.66, 2.08)	-0.09 (-0.40, 0.22)

CI, confidence interval; NSAID, Non-Steroidal Anti-Inflammatory Drug; Avg no., Average number

© 2021 Keshwani S et al. *JAMA Network Open*.

eFigure. Number of Days' Supply for Oral NSAID Users per Month

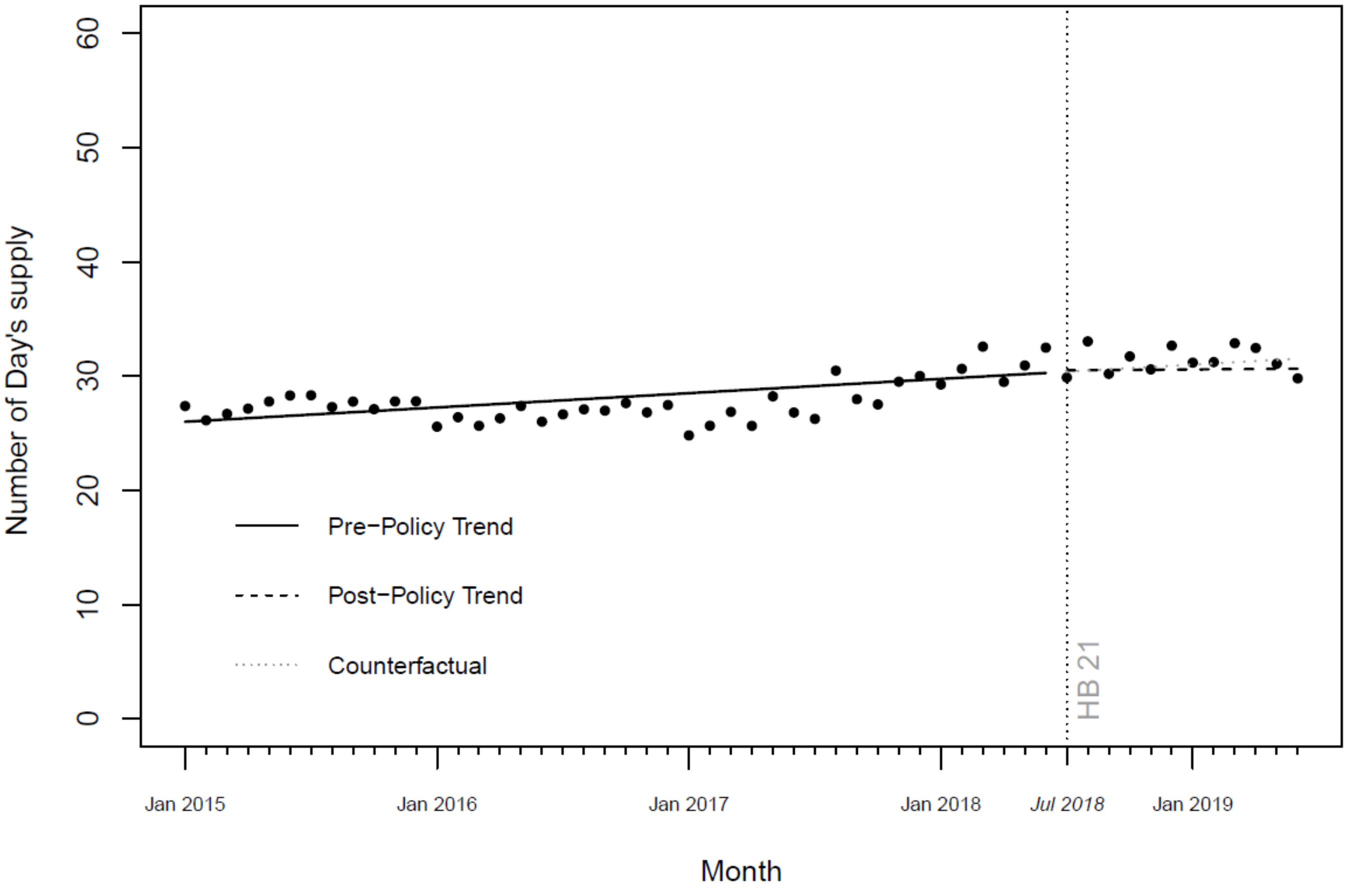

© 2021 Keshwani S et al. *JAMA Network Open*.
